# Molecular sp^3^‑like Reactivity of Metastable Au_4_Si near Its Deep Eutectic Point Enables Low-Temperature SiC Formation

**DOI:** 10.1021/acs.jpclett.6c00179

**Published:** 2026-03-11

**Authors:** Jhong-Ren Huang, Yi-Hsin Liu, Satoshi Kameoka, Lu-Sheng Hong

**Affiliations:** † Department of Mechanic Engineering, 34878 National Taiwan University of Science and Technology, No. 43, Sec. 4, Keelung Rd., Da’an Dist., Taipei City 106335, Taiwan; ‡ Department of Chemical Engineering, 34878 National Taiwan University of Science and Technology, No. 43, Sec. 4, Keelung Rd., Da’an Dist., Taipei City 106335, Taiwan; § Institute of Multidisciplinary Research for Advanced Materials, Tohoku University, 41 Kawauchi, Aoba-ku, Sendai 980-8576, Japan

## Abstract

Metastable states near deep eutectic points are typically regarded as transient intermediates preceding phase separation, yet their potential chemical reactivity remains largely unexplored. Here, we demonstrate that metastable Au–Si bonding configurations derived from Au_4_Si near its deep eutectic temperature exhibit molecule-like reactivity associated with an sp^3^-like local bonding environment, enabling direct Si–C bond formation at temperatures as low as 636 K. Using a high-vacuum coevaporation platform, Au–Si species generated during coevaporation react with carbon clusters to produce SiC accompanied by Au segregation, whereas elemental Si under identical conditions remains chemically inert. Raman spectroscopy and X-ray photoelectron spectroscopy reveal that SiC formation occurs only within a narrow temperature window centered at the eutectic point and displays nonmonotonic temperature dependence inconsistent with conventional catalytic or vapor–liquid–solid mechanisms. These results provide experimental evidence that eutectic metastable bonding configurations can transiently adopt molecule-like characteristics, thereby enabling unconventional low-temperature reaction pathways in metal–semiconductor systems.

## Graphical abstract

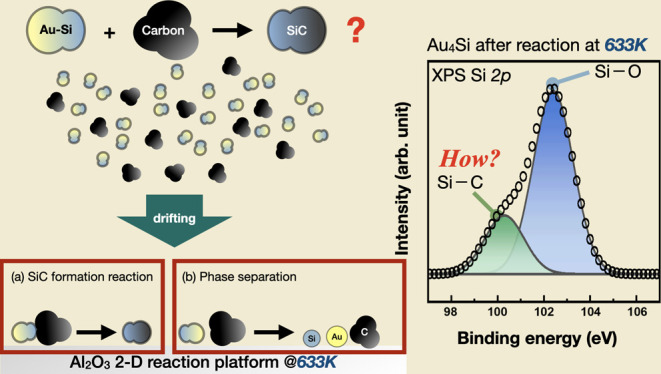

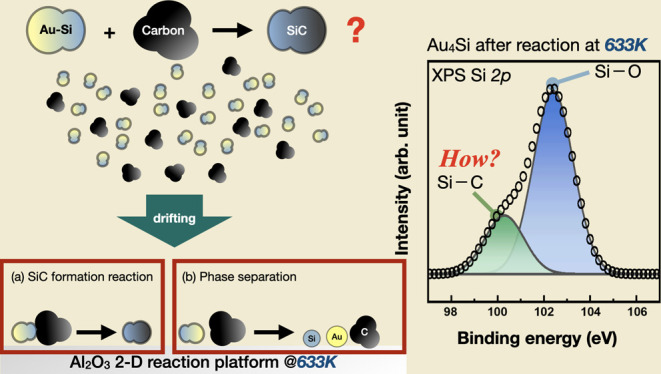

The gold–silicon (Au–Si) binary system is a prototypical deep-eutectic alloy, characterized by a pronounced melting-point depression and immiscibility below the eutectic temperature of 636 K.^1^ Owing to this anomalous thermodynamic behavior, Au–Si has long served as a model system for studying supercooling, eutectic solidification, and nonequilibrium phase formation.^2–8^ Numerous studies have reported the emergence of transient metastable phases near the eutectic point, which are commonly interpreted as intermediates arising during the evolution toward thermodynamic equilibrium.^8–17^

In particular, several experimental and theoretical investigations have established that the Au–Si alloy exhibits distinct crystalline ordering near the eutectic composition of Au_81_Si_19_, rather than a simple liquid mixture of elemental Au and Si.^12–21^ Fast calorimetry measurements revealed that the Au–Si eutectic system undergoes multiple metastable-to-stable phase transitions during melting, within which a single γ-like phase exists over a narrow temperature window near the eutectic point,^17^ while complementary theoretical and spectroscopic studies demonstrated that the metastable Au_4_Si phase adopts an ordered atomic arrangement with Si atoms locally coordinated by Au atoms in a manner consistent with sp^3^-like directional bonding motifs in crystalline silicon.^12,20,21^ Taken together, these observations suggest that Au_4_Si near the eutectic point can transiently access an ordered, molecular-like bonding configuration similar to that of molecular silicon precursors such as silane (SiH_4_).^22–24^

However, whether such metastable, molecule-like bonding configurations can manifest chemical reactivity, rather than merely structural ordering, has remained largely unexplored. This consideration raises the possibility that the sp^3^-like bonding environment in metastable Au_4_Si may enable chemical transformations that are otherwise inaccessible to elemental silicon. In this work, we examine this possibility by probing the interaction between metastable Au_4_Si and carbon near the eutectic temperature using a high-vacuum coevaporation platform that confines reactions to a quasi-two-dimensional surface. By combining temperature-resolved electron microscopy with Raman and X-ray photoelectron spectroscopy, we demonstrate that Si–C bond formation occurs exclusively within a narrow eutectic-centered temperature window and deviates from conventional Arrhenius or vapor–liquid–solid reaction pathways. These findings establish a direct link between eutectic metastability and emergent chemical reactivity, providing new insight into unconventional low-temperature reaction pathways in metal–semiconductor systems.

Au_4_Si alloys with a nominal composition of Au_80_Si_20_ were prepared from high-purity Au and Si (99.99%) and used as silicon sources. Commercial carbon black (99.999%) served as the carbon precursor. Coevaporation experiments were conducted in a high-vacuum chamber evacuated to a base pressure of ∼10^–8^ Torr prior to deposition. The working pressure during coevaporation was maintained at ∼4 × 10^–6^ Torr. Under these conditions, the partial pressure of residual oxygen is estimated to be 2 orders of magnitude smaller than the total deposition flux. Depositions were performed on sapphire substrates (1 × 1 cm^2^) serving as quasi-two-dimensional reaction platforms. Au_4_Si (or elemental Si for reference experiments) and carbon sources were simultaneously evaporated by resistive heating and electron-beam evaporation, respectively. Deposition rates were independently monitored by quartz crystal microbalances and maintained at a Si:C atomic ratio of 1:1, with a total nominal thickness of 25 nm. Under these vacuum conditions, the mean free path of the vapor species exceeded the source–substrate distance, effectively suppressing gas-phase collisions and confining reactions to the substrate surface. Substrate temperatures were controlled between 533 and 653 K, spanning the solid and liquid regimes of the Au–Si system, with particular focus on the eutectic temperature near 636 K. Surface morphology was examined by scanning electron microscopy to identify phase segregation behavior. Raman spectroscopy was employed to probe Si–C bond formation, while X-ray photoelectron spectroscopy was used to quantify the SiC conversion via analysis of the Si 2p core-level spectra. Additional experimental details are provided in the Supporting Information.

Figure 1 presents SEM images of as-deposited samples prepared at substrate temperatures ranging from 533 to 653 K, spanning the solid and liquid regimes of the Au–Si system. SEM observations were carried out in secondary electron (SE) mode to examine the surface morphology and segregation behavior. The brighter contrast is attributed to Au-rich particles, whereas the darker regions correspond to exposed sapphire substrate. This assignment is supported by AES elemental mapping (Figure S2), which confirms that the bright nanoparticles correspond to Au-rich domains. The particle morphology exhibits a pronounced dependence on the substrate temperature. Below the eutectic temperature (623 K, Figure 1b), relatively compact features are observed, consistent with segregation behavior in the Au–Si system below the eutectic temperature. Near the eutectic point (638 K, Figure 1c), irregularly shaped nanoparticles emerge, closely resembling morphologies previously reported for solidification near the Au–Si eutectic composition.^17,25^ At higher temperatures (653 K, Figure 1d), increased surface porosity becomes evident, suggesting partial re-evaporation of Au–Si species under overheated conditions. Importantly, a sharp morphological transition is observed only within a narrow temperature window centered near the eutectic point, consistent with prior reports of eutectic-related morphological transitions.^26^ While SEM does not provide direct information on chemical bonding or reaction products, the emergence of this distinct morphological regime near the eutectic temperature delineates the temperature range of interest and motivates subsequent spectroscopic analyses to probe the underlying chemical processes.

**Figure 1:**
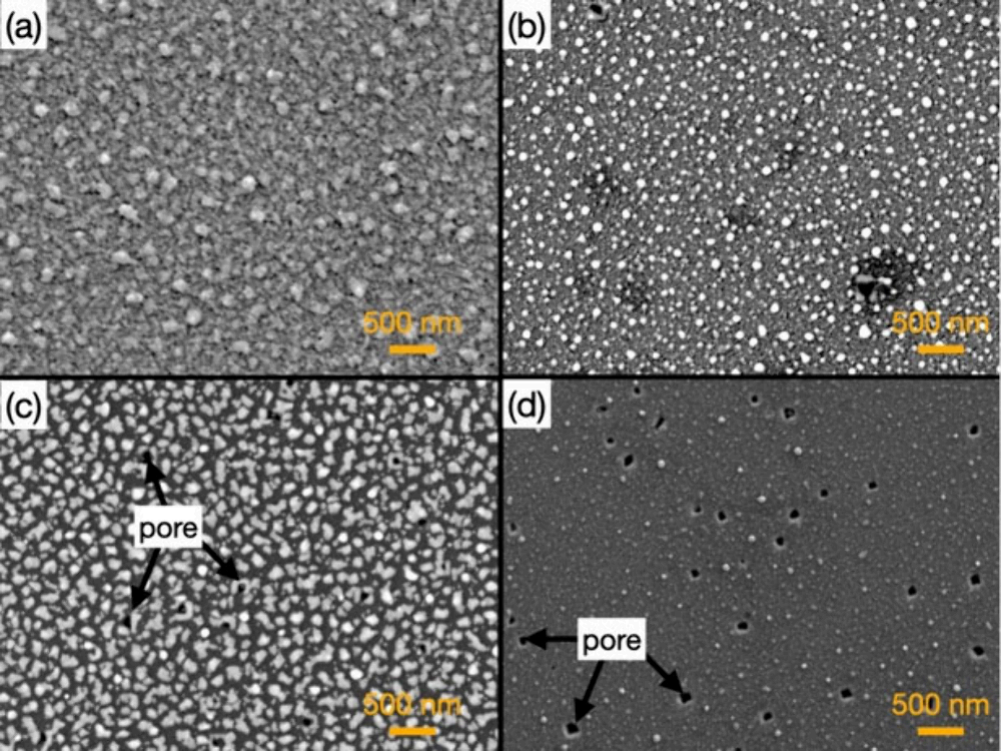
Secondary electron (SE) SEM images of as-deposited samples prepared on Al_2_O_3_ (sapphire) substrates using Au_4_Si and carbon as precursors at substrate temperatures of (a) 533, (b) 623, (c) 638, and (d) 653 K. The darker background corresponds to the exposed sapphire substrate, while the brighter particles correspond to Au-rich segregated domains.

To elucidate the chemical origin of the morphological transitions observed near the eutectic point, Raman spectroscopy was employed to probe the bonding configurations of the deposited species. Raman spectra were acquired from samples prepared using Au_4_Si and carbon as precursors on sapphire substrates, together with a reference sample deposited using elemental Si and carbon at 673 K (Figure 2). Pronounced Si–C vibrational features are observed only for samples prepared with Au_4_Si precursors and only when the substrate temperature exceeds 623 K. No corresponding Si–C signals are detected in samples deposited using elemental Si under identical conditions. The dominant Si–C-related Raman bands appear in the range of 580–760 cm^–1^, distinct from the characteristic amorphous SiC band near 870 cm^–1^.^27^ This spectral signature is consistent with structurally distorted or kinetically constrained SiC formation rather than well-relaxed amorphous phases. The concurrent observation of Si–C vibrational features and Au-rich precipitates suggests that SiC formation is accompanied by Au segregation, consistent with chemical segregation in the Au–Si system during deposition. Taken together, SEM delineates a narrow temperature window centered near the eutectic point, in which a distinct metastable Au–Si state is manifested, while Raman spectroscopy provides direct evidence that Si–C bond formation is strongly temperature-selective within this window. These observations support a scenario in which metastable Au_4_Si-derived species react with carbon clusters on the quasi-two-dimensional reaction platform, leading to SiC formation while Au segregates into precipitates.

**Figure 2:**
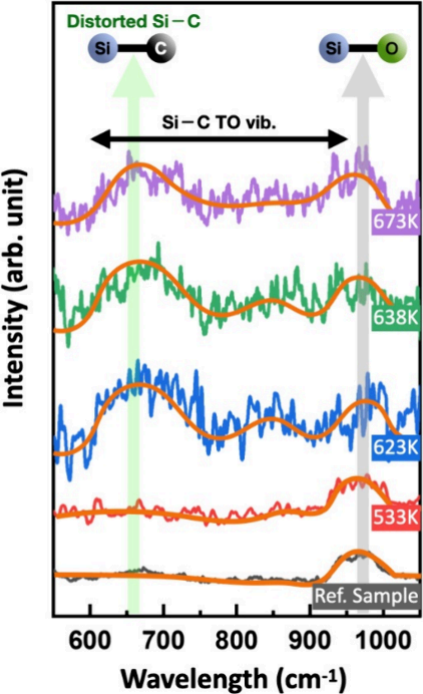
Raman spectra of as-deposited samples prepared using Au_4_Si and carbon precursors at different substrate temperatures, together with a reference sample prepared using elemental Si and carbon at 673 K.

Figure 3 presents representative Si 2p XPS spectra of samples prepared by coevaporation of Au_4_Si and carbon at different substrate temperatures (full data sets are provided in Figure S3). At 533 K (Figure 3a), no Si–C component at ∼100.3 eV is detected,^28^ consistent with the absence of Si–C vibrational features in Raman spectroscopy. Instead, the Si 2p signal is dominated by oxidized species (Si–O at ∼103 eV), which is attributed primarily to postdeposition ambient oxidation of unreacted Si precipitates, indicating that Si–C bond formation is negligible at this temperature. In this low-temperature regime, Au–Si species are expected to undergo phase separation rather than an effective interfacial reaction with carbon, consistent with the compact morphology observed in Figure 1a. As the substrate temperature approaches the eutectic point, a distinct Si–C component emerges in the Si 2p spectra (Figure 3b–d). Correspondingly, the C 1s spectra (Figure S4) show a C–Si feature at ∼283.2 eV^28^ for samples prepared using Au_4_Si, whereas this signal is absent for the reference sample prepared using elemental Si under otherwise identical conditions. The combined XPS and Raman results therefore establish that Si–C bond formation is strongly temperature-selective and occurs only within a narrow temperature window centered near the eutectic point. The temperature-selective emergence of the Si–C component, in contrast to the broad presence of Si–O signals, indicates that oxygen does not govern the reaction pathway but instead reflects postdeposition oxidation of residual Si. These observations are consistent with the notion that the bonding environment in metastable Au–Si configurations near the eutectic point differs from that of elemental Si,^20,21^ enabling a reaction pathway that is not accessible for Si and carbon in direct contact within the same temperature regime.^29,30^ While the microscopic mechanism remains to be clarified, the emergence of Si–C bonding near the eutectic point provides experimental support for eutectic-state reactivity beyond conventional thermal activation; notably, even reactions between active carbon sources such as acetylene on Si surfaces often require substantially higher temperatures to proceed.^30^

**Figure 3:**
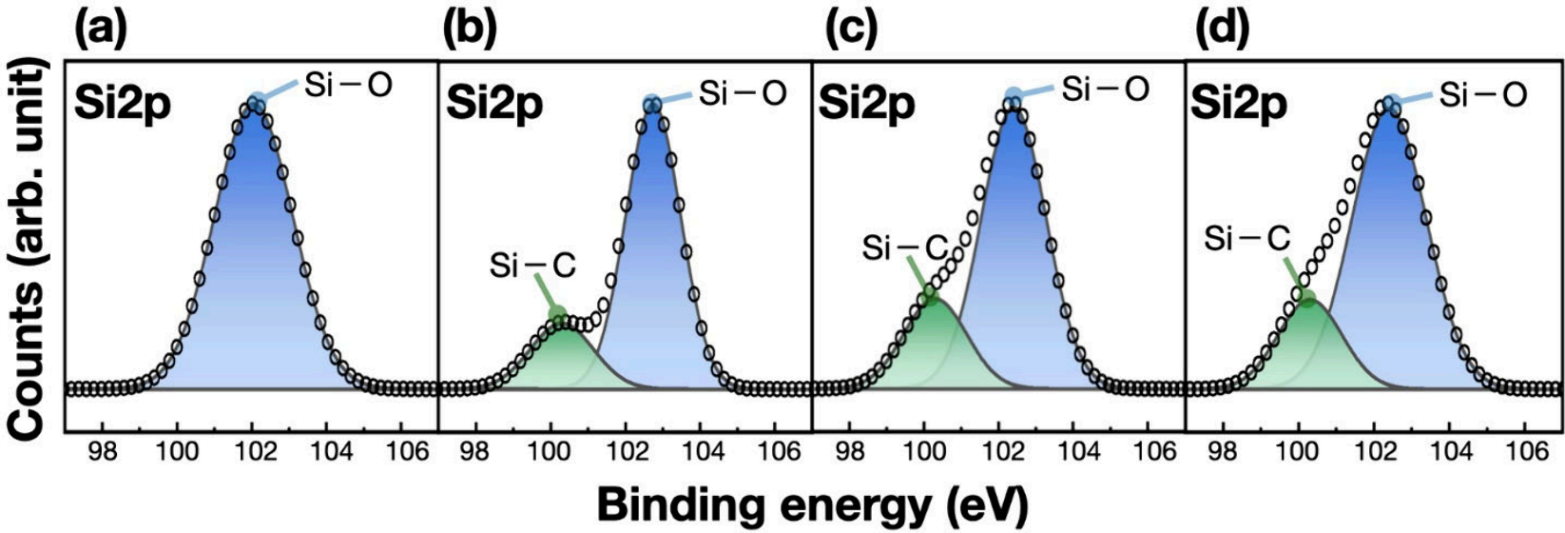
Si 2p XPS spectra of samples prepared by coevaporation of Au_4_Si and carbon at substrate temperatures of (a) 533, (b) 623, (c) 638, and (d) 653 K. The deposition duration was 10 min.

To quantify the relationship between the eutectic-centered regime and the extent of SiC formation, the conversion ratio was evaluated by integrating the Si–C component in the Si 2p spectra, as described below.

Figure 4 summarizes the temperature dependence of the SiC conversion ratio extracted from the Si 2p XPS spectra. The conversion reaches a maximum value of approximately 25% at ∼636 K, which closely coincides with the eutectic temperature of the Au–Si system. Notably, increasing the substrate temperature above the eutectic point does not enhance the conversion ratio. This nonmonotonic temperature dependence deviates from conventional Arrhenius-type kinetics and is inconsistent with a vapor–liquid–solid (VLS) catalytic mechanism, in which high temperatures would normally promote reaction efficiency.^31^ Instead, the conversion appears confined to a narrow temperature window centered at the eutectic point, indicating that the chemical activity is governed by a metastability-defined regime rather than by thermal activation alone.

**Figure 4:**
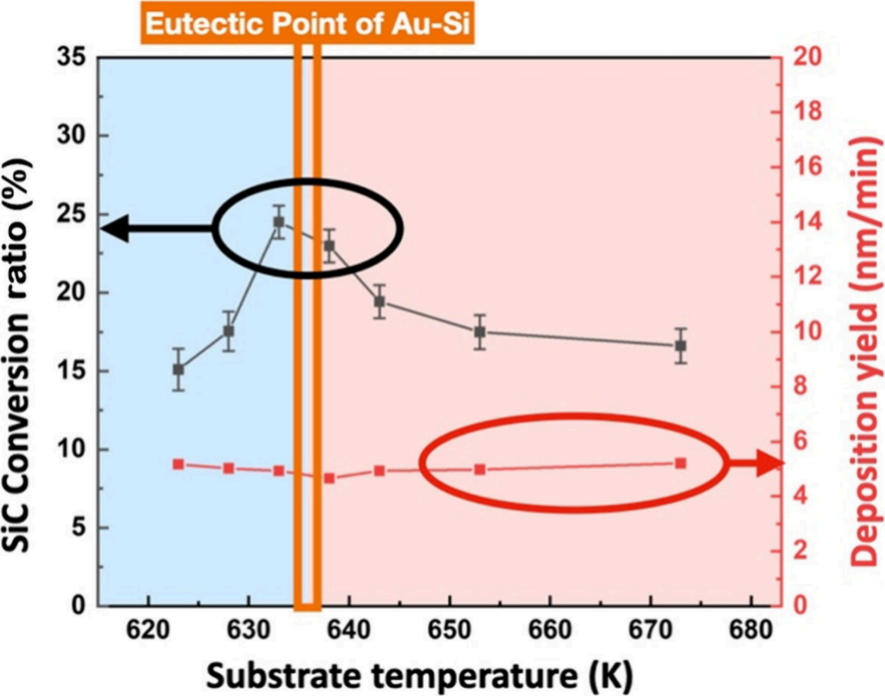
Temperature dependence of the SiC conversion ratio extracted from the Si 2p XPS spectra.

Conventional approaches to SiC formation, such as plasma-assisted CVD and molecular precursor routes, typically rely on gas-phase activation or externally generated energetic species. In contrast, the present system operates under high-vacuum, surface-confined conditions without plasma assistance or reactive gaseous precursors. Notably, Si–C bond formation is observed at ∼636 K within a narrow temperature window centered on the eutectic region and is associated with transient, metastable Au–Si bonding configurations rather than external activation. While the conversion remains partial and surface-confined, consistent with the nonequilibrium and surface-limited nature of the process, the results reveal a distinct reactivity emerging near the eutectic point.

While the present results establish the temperature selectivity and extent of SiC formation, the microscopic reaction pathway remains to be elucidated. Based on the observed eutectic-centered reactivity and prior structural studies of Au_4_Si,^20,21^ the following discussion is intended as a conceptual framework rather than a definitive mechanism. From this perspective, metastable Au–Si configurations inferred to exhibit an sp^3^-like local bonding environment near the eutectic point may be conceptually analogous to electronically activated silicon states invoked in low-temperature SiC growth using molecular precursors (e.g., SiH_4_). This analogy does not imply an identical pathway but provides an intuitive basis for understanding how transient, molecule-like bonding configurations could facilitate Si–C bond formation under otherwise inaccessible conditions.

Within this framework, Si–C bond formation may arise from the transient accessibility of reactive Au–Si bonding states near the eutectic point, potentially associated with local coordination rearrangements during phase separation. Such effects could lower the kinetic barrier toward formation of the thermodynamically favored SiC phase and thereby give rise to the observed eutectic-selective reactivity.

Importantly, Au_4_Si serves here as a model system to illustrate the concept of “eutectic-centered reactivity”. The present findings suggest that transient metastable bonding configurations emerging near deep eutectic points may not be unique to the Au–Si alloy and could be generalized to other alloy systems exhibiting analogous nonequilibrium bonding characteristics.

In summary, we investigated the deposition behavior and reactivity of Au–Si vapor species derived from Au_4_Si with carbon clusters on sapphire substrates over a temperature range spanning the Au–Si eutectic point. Raman spectroscopy and X-ray photoelectron spectroscopy demonstrate that Si–C bond formation occurs exclusively within a narrow temperature window centered near the eutectic temperature, with a maximum SiC conversion of approximately 25% at ∼636 K. The absence of reactivity in elemental Si reference samples and the deviation from Arrhenius-type behavior indicate that SiC formation is not governed by conventional catalytic or thermally activated mechanisms. Instead, the results provide experimental evidence that eutectic metastable bonding states can enable unconventional chemical reactivity, offering new insight into low-temperature solid-state reaction pathways in metal–semiconductor systems.

## Supplementary Material



## Notes

Experimental details of the high-vacuum coevaporation system for Au_4_Si and C, AES elemental mapping of Au, Si, and C after codeposition, temperature-dependent XPS analysis of Si–C bond formation, SiC conversion ratio estimation (PDF)
