# Supplementary material for: Molecular sp3‑like Reactivity of Metastable Au4Si near Its Deep Eutectic Point Enables Low-Temperature SiC Formation
Source: J Phys Chem Lett. 2026 Mar 11;17(12):3510–4. doi: 10.1021/acs.jpclett.6c00179 (PMC13034462; doi:10.1021/acs.jpclett.6c00179)
Supplement: Supplementary file 1 [file jz6c00179_si_001.pdf]

Supporting Information

Molecular sp^3-Like Reactivity of Metastable Au_4Si Near Its Deep Eutectic Point Enables Low-Temperature SiC Formation

Jhong-Ren Huang[a], Yi-Hsin Liu[b], Satoshi Kameoka[c], Lu-Sheng Hong[b] *

[a]*Department of Mechanic Engineering, National Taiwan University of Science and Technology, No. 43, Sec. 4, Keelung Rd., Da'an Dist., Taipei City 106335, Taiwan.*

[b]*Department of Chemical Engineering, National Taiwan University of Science and Technology, No. 43, Sec. 4, Keelung Rd., Da'an Dist., Taipei City 106335, Taiwan.*

[c]*Institute of Multidisciplinary Research for Advanced Materials, Tohoku University, 41 Kawauchi, Aoba-ku, Sendai, 980-8576 Japan.*

Experimental details of the high-vacuum co-evaporation system for Au_4Si and C

A high-vacuum co-evaporation system (HVCS) was employed for the simultaneous deposition of silicon (Si) and carbon (C) species. As illustrated in Figure S1, the system consists of two independent evaporation sources: a resistively heated boat for Au_4Si (or elemental Si) evaporation and an electron-beam (e-beam) evaporator used for carbon black. The e-beam stage is interchangeable, allowing source replacement under vacuum without breaking chamber conditions. Prior to deposition, all shutters were closed and the chamber was evacuated to a base pressure of approximately 4×10^{-7} Torr. The pressure was further reduced to $\sim 10^{-8}$ Torr via titanium gettering using e-beam evaporation. After gettering, the titanium source was replaced

with carbon black, and carbon evaporation was initiated once a stable deposition rate was achieved, as monitored in situ by a quartz crystal microbalance (QCM). In parallel, the Au_4Si source was resistively heated to induce melting. The evaporation flux was adjusted to maintain a nominal atomic ratio of C:Si = 1:1. After stabilization of both evaporation rates, co-deposition was initiated by opening all shutters onto rotating substrates. The average deposition rate was ~ 5 nm min^{-1}, and deposition proceeded for ~5 min, yielding a nominal film thickness of approximately 25 nm as measured by QCM. Experimental parameters are summarized in Table S1. During co-evaporation, the working pressure was maintained at ~ 4.4×10^{-6} Torr. Under these conditions, the mean free path of vapor species extends to several tens of meters, far exceeding the source–substrate distance, thereby minimizing gas-phase collisions and confining reactions to the substrate surface.

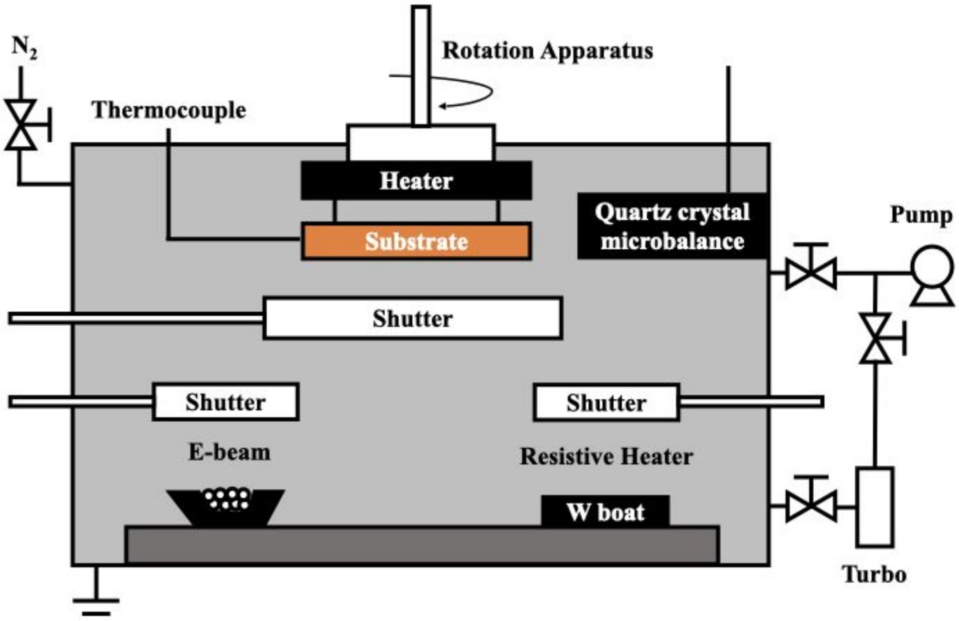

Figure S1. Schematic illustration of the high-vacuum co-evaporation system equipped with a resistive heater and an electron-beam evaporation source.

Table S1. Experimental parameters for Au_4Si/C co-evaporation (Samples A–G) and the reference Si/C co-evaporation sample (R).

**Sample**	**Si source**	**C source**	**Substrate temperature (K)**	**Background pressure (Torr)**	**Working pressure (Torr)**	**Deposition rate (nm min^{-1})**
A	Au_4Si	carbon black	593	4.6×10^{-7}	4.1×10^{-6}	4.8
B	Au_4Si	carbon black	628	3.8×10^{-7}	4.7×10^{-6}	5.2
C	Au_4Si	carbon black	633	5.0×10^{-7}	4.3×10^{-6}	5.0
D	Au_4Si	carbon black	638	3.2×10^{-7}	3.9×10^{-6}	4.9
E	Au_4Si	carbon black	643	2.9×10^{-7}	4.4×10^{-6}	4.7
F	Au_4Si	carbon black	653	3.6×10^{-7}	5.1×10^{-6}	4.9
G	Au_4Si	carbon black	673	3.7×10^{-7}	4.6×10^{-6}	5.2
R	Si	carbon black	673	4.0×10^{-7}	3.7×10^{-6}	5.0

AES elemental mapping of Au, Si, and C after co-deposition

Figure S2 presents Auger electron spectroscopy (AES) elemental mapping of Au, Si, and C for a representative sample deposited near the eutectic temperature, together with the corresponding SEM image (Figure S2a). The Au map (Figure S2c) shows strong localization within the large bright particles observed in SEM, confirming segregation into Au-rich domains. In contrast, the Si map (Figure S2d) exhibits enhanced intensity in the surrounding matrix and reduced signal within Au-rich particles, indicating complementary spatial distribution of Au and Si. The carbon map (Figure S2b) displays weaker overall contrast, consistent with the lower Auger yield and surface sensitivity of carbon in AES measurements. Nevertheless, reduced carbon intensity over Au-rich particles and relatively higher signal in the surrounding matrix can be discerned. Although the contrast is modest, this spatial trend suggests preferential association of carbon with Si-containing regions rather than Au-rich domains. Owing to the limited sensitivity of AES for light elements, the carbon distribution is interpreted qualitatively. The maps therefore confirm Au–Si segregation and indicate co-localization of carbon with Si-rich regions after co-evaporation.

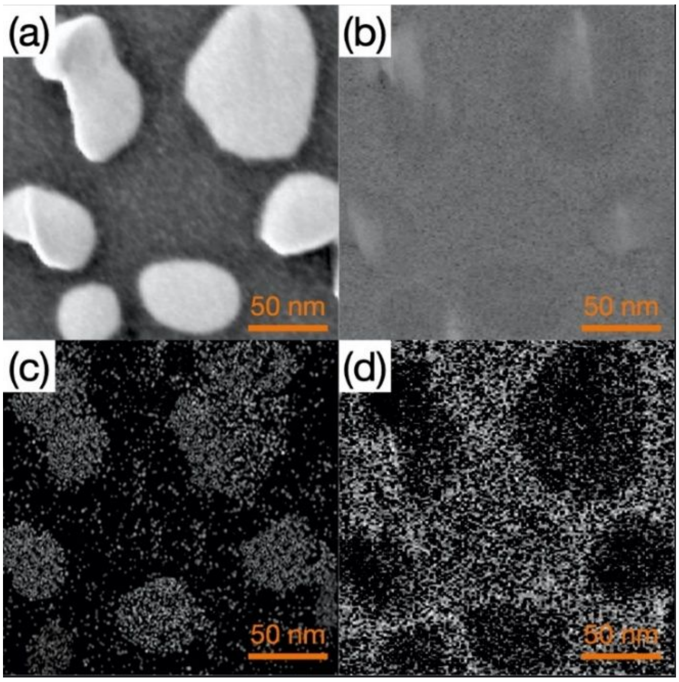

Figure S2. Representative Auger electron spectroscopy (AES) elemental mapping of an as-deposited sample prepared on an Al_2O_3 substrate using Au_4Si as the Si precursor at 623 K: (a) secondary electron image, (b) C map, (c) Au map, and (d) Si map.

Temperature-dependent XPS analysis of Si–C bond formation

Figure S3 presents representative Si 2p XPS spectra of samples prepared using Au_4Si as the Si precursor at substrate temperatures ranging from 593 to 673 K, together with a reference sample prepared using elemental Si at 673 K. At lower substrate temperatures (593–623 K), the Si 2p spectra are dominated by a component centered near ~103 eV, which is attributed to oxidized Si species (Si–O). In this temperature range, the Si–C component at ~100.3 eV is absent or negligible, consistent with the lack of Si–C vibrational features in Raman spectroscopy and indicating that Si–C bond formation is suppressed below the eutectic region. As the substrate temperature approaches the Au–Si eutectic temperature (~636 K), a distinct Si–C component emerges near 100.3 eV (see 628–653 K). The relative intensity of this component reaches a maximum in the vicinity of the eutectic temperature and decreases again at higher temperatures (e.g., 673 K), demonstrating a non-monotonic temperature

dependence. In contrast, the reference sample prepared using elemental Si at 673 K does not exhibit a discernible Si–C component under otherwise identical deposition conditions. This comparison confirms that the observed Si–C bond formation is not a general consequence of thermal activation but is specifically associated with the Au_4Si-derived system.

Figure S4 compares the C 1s XPS spectra of samples prepared at 673 K using (a) elemental Si and carbon, and (b) Au_4Si and carbon. For the elemental Si + C sample (Figure S4a), the C 1s spectrum is dominated by a peak near 284.5 eV, characteristic of C–C bonding, with no discernible component at ~283.2 eV corresponding to Si–C bonding. This result indicates that direct Si–C bond formation does not occur under these conditions when elemental Si is employed as the precursor. In contrast, the Au_4Si + C sample (Figure S4b) exhibits an additional component near ~283.2 eV, which is assigned to C–Si bonding. Although the overall signal-to-noise ratio of the C 1s spectra is lower than that of the Si 2p region due to the thin-film geometry and intrinsic surface sensitivity of XPS, the emergence of this lower binding energy component is clearly resolved. The presence of this feature is consistent with Si–C bond formation and is in good agreement with the corresponding Si 2p analysis shown in Figure S3.

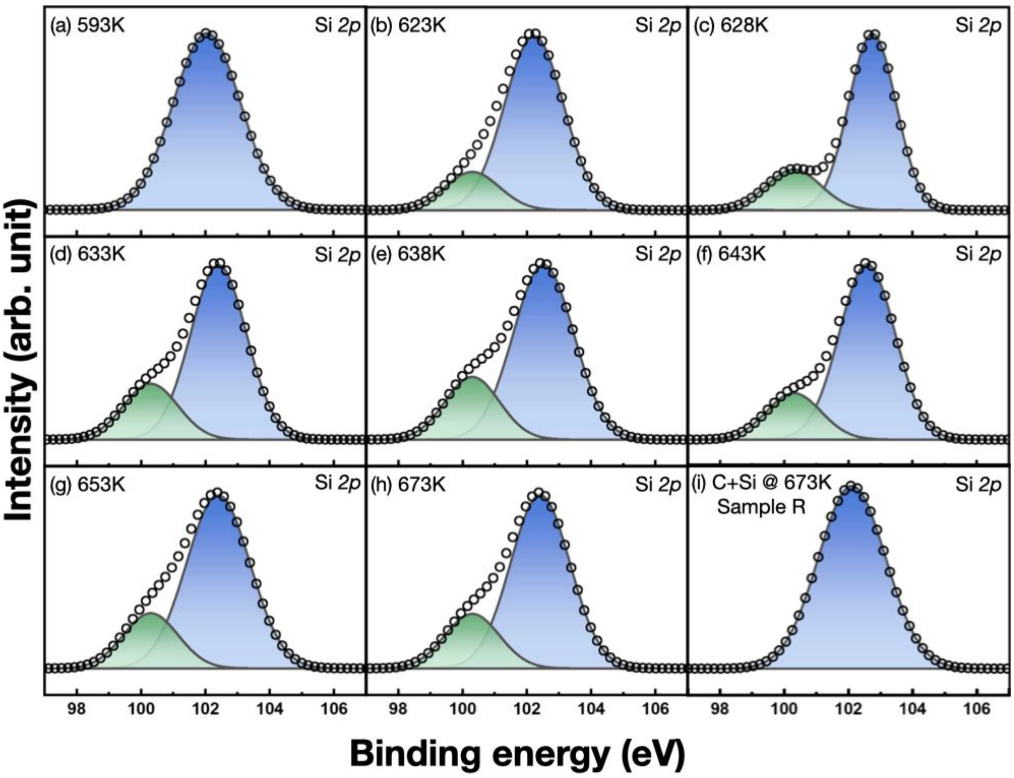

Figure S3. Representative XPS Si 2p spectra of samples prepared using Au_4Si as Si precursor at substrate temperatures of (a) 593 K, (b) 623 K, (c) 628 K, (d) 633 K, (e) 638 K, (f) 643 K, (g) 653 K, and (h) 673 K, together with (i) a reference sample prepared using elemental Si at 673 K.

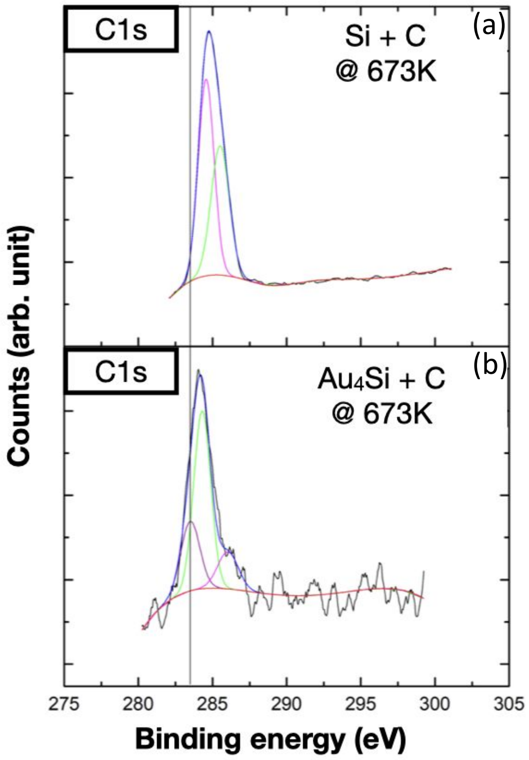

Figure S4. XPS C 1s spectra of samples prepared at 673 K using different Si sources: (a) elemental Si and carbon and (b) Au_4Si and carbon.

SiC conversion ratio estimation

The SiC conversion ratio (x) was estimated from the Si 2p X-ray photoelectron spectroscopy (XPS) spectra based on the simplified reaction scheme:

$$Au_4Si + C \rightarrow x SiC + 4Au + (1 - x)Si, \ 0 \leq x \leq 1. \qquad (1)$$

Following co-deposition, two Si-containing species are expected: SiC and residual Si. Because elemental Si is highly susceptible to oxidation upon exposure to ambient conditions, any unreacted Si is assumed to convert predominantly into silicon oxide (SiO_x). Accordingly, the Si–O component is treated here as an indirect measure of residual unreacted Si for the purpose of estimating the SiC conversion ratio, and this assumption is explicitly acknowledged in the analysis. Under this assumption, the relative amounts of the Si–C and Si–O components in the Si 2p spectra provide an indirect measure of the extent of SiC formation. Although the evaporation fluxes were

adjusted to maintain a nominal atomic ratio of C:Si = 1:1, it should be noted that carbon deposited under these conditions tends to form clusters on the substrate surface due to its limited surface mobility. As a result, not all supplied carbon may be equally accessible for interfacial reaction with Au_4Si-derived species. Therefore, the measured conversion ratio reflects the effective extent of Si–C bond formation under kinetically constrained surface conditions rather than the total stoichiometric carbon supply. The SiC conversion ratio was calculated as:

$$x = \frac{A_{\mathrm{Si-C}}}{A_{\mathrm{Si-C}} + A_{\mathrm{Si-O}}} \quad (2),$$

where $A_{\mathrm{Si-C}}$ and $A_{\mathrm{Si-O}}$ represent the integrated peak areas of Si–C and Si–O components, respectively, obtained from deconvolution of the Si 2p spectra. Peak fitting was performed with constrained full width at half maximum (FWHM) values of 1.5–1.8 eV. Gaussian line shapes were employed for the SiO_x component. The integrated areas extracted from the fitted spectra were used to calculate the conversion ratio according to Eq. (2). The resulting values, together with their standard deviations, are plotted as a function of substrate temperature in Figure 4.
